# Prediction of metabolic syndrome based on sleep and work-related risk factors using an artificial neural network

**DOI:** 10.1186/s12902-020-00645-x

**Published:** 2020-11-12

**Authors:** Meysam Eyvazlou, Mahdi Hosseinpouri, Hamidreza Mokarami, Vahid Gharibi, Mehdi Jahangiri, Rosanna Cousins, Hossein-Ali Nikbakht, Abdullah Barkhordari

**Affiliations:** 1 Department of Occupational Health Engineering, School of Public Health, Tehran University of Medical Sciences, Tehran, Iran; 2 Center of Planning, Budgeting and Performance Evaluation, Department of Environment, Tehran, Iran; 3 Department of Ergonomics, School of Public Health, Shiraz University of Medical Sciences, Shiraz, Iran; 4 Department of Occupational Health, School of Health, Shiraz University of Medical Sciences, Shiraz, Iran; 5 Department of Psychology, Liverpool Hope University, Liverpool, UK; 6 Social Determinants of Health Research Center, Health Research Institute, Department of Biostatistics & Epidemiology, Faculty of Medicine, Babol University of Medical Sciences, Babol, Iran; 7 Department of Occupational Health, School of Public Health, Shahroud University of Medical Sciences, Shahroud, Iran

**Keywords:** Metabolic syndrome, Work-related stressors, Obstructive sleep apnea, Workplace, Modelling

## Abstract

**Background:**

Metabolic syndrome (MetS) is a major public health concern due to its high prevalence and association with heart disease and diabetes. Artificial neural networks (ANN) are emerging as a reliable means of modelling relationships towards understanding complex illness situations such as MetS. Using ANN, this research sought to clarify predictors of metabolic syndrome (MetS) in a working age population.

**Methods:**

Four hundred sixty-eight employees of an oil refinery in Iran consented to providing anthropometric and biochemical measurements, and survey data pertaining to lifestyle, work-related stressors and sleep variables. National Cholesterol Education Programme Adult Treatment Panel ІІI criteria was used for determining MetS status. The Management Standards Indicator Tool and STOP-BANG questionnaire were used to measure work-related stress and obstructive sleep apnoea respectively. With 17 input variables, multilayer perceptron was used to develop ANNs in 16 rounds of learning. ANNs were compared to logistic regression models using the mean squared error criterion for validation.

**Results:**

Sex, age, exercise habit, smoking, high risk of obstructive sleep apnoea, and work-related stressors, particularly *Role,* all significantly affected the odds of MetS, but shiftworking did not. Prediction accuracy for an ANN using two hidden layers and all available input variables was 89%, compared to 72% for the logistic regression model. Sensitivity was 82.5% for ANN compared to 67.5% for the logistic regression, while specificities were 92.2 and 74% respectively.

**Conclusions:**

Our analyses indicate that ANN models which include psychosocial stressors and sleep variables as well as biomedical and clinical variables perform well in predicting MetS. The findings can be helpful in designing preventative strategies to reduce the cost of healthcare associated with MetS in the workplace.

**Supplementary Information:**

**Supplementary information** accompanies this paper at 10.1186/s12902-020-00645-x.

## Background

Metabolic syndrome (MetS) is a clustering of interrelated non-communicable factors that is useful for identifying individuals with an increased risk of developing cardiovascular diseases, and type 2 diabetes mellitus (T2DM) [1, 2]. To reduce high prevalence rates, and improve the health of populations, better methods of predicting MetS are urgently needed. The greatest impact of MetS is seen in productive working age populations aged 45–64 years [1, 3, 4], even though the burden of the chronic diseases is mainly over the age of 65 years in most developed countries [3]. High prevalence rates of MetS have been found in population studies in Iran [1, 4], and notably, a comprehensive nationwide study reported age-standardised prevalence rates of 3024 participants aged 25–64 years as 34.7%, with significantly higher rates in females than males in all age categories [4].

Work-related risk factors and personal lifestyle habits can contribute to the development of MetS [5, 6]. Evidence that work-related stress (WRS) induces MetS is found in a prospective study of 234 Police Officers in Italy [7], and 30 years of Whitehall II studies in the UK reliably indicate that WRS predicts CVD, although the link with T2DM is less consistent [8–10]. The UK’s Health and Safety Executive (HSE) developed an approach based on Management Standards [8, 11] to deal with stress at work. The seven-factor Management Standards Indicator Tool (MSIT) [11] is reliable for identifying risks for WRS [12–14]. It has been translated into many languages including Persian [14] making it an appropriate measure for inclusion in a comprehensive examination of predictors of MetS.

There is also evidence that shiftwork has significant effects on MetS [6]. Shiftwork is driven by economic efficiency, which generally outshines evidence that disturbances to circadian rhythms and normal sleep patterns can have negative health consequences. Similarly, obstructive sleep apnoea (OSA), a sleep-related breathing disorder, has been implicated in the development of MetS [15, 16]. Most metabolic syndrome components – central obesity, elevated plasma glucose, dyslipidaemia and high blood pressure – are individually related to OSA, in line with severity of OSA [17], with obesity and abdominal fat accumulation known to be key factors in developing OSA [1, 18]. Furthermore, upper airway collapse and intermittent hypoxia increases glucose intolerance, which contributes to the pathogenesis in co-morbidities, including MetS [18]. Thus, it is important that models to predict MetS in any working population should include data on sleep disorders and work schedules.

To our knowledge, no study to date has investigated the simultaneous effects of work-related stressors and sleep disturbance on MetS, using National Cholesterol Education Program Adult Treatment Panel ІІI (ATPІІІ) criteria [2]. The present study was designed to examine this gap. The study focused on modelling MetS in a community workforce in Iran incorporating feedforward multilayer perceptron [19] artificial neural networks (ANN) with resilient backpropagation as the training algorithm. This algorithm is fast and does not require as much tuning as classic backpropagation [20]. ANN are a powerful tool for recognizing complex functional relationships between covariates and response variables via a learning process [20] and are particularly suitable for prediction of medical diagnoses, including diabetes and pre-diabetes [21–27]. After training, an ANN system can be applied to predict the output from a given input of new data. There is evidence that ANN are better predictive models than linear models in several clinical fields [28–31], including a demonstration that ANN are superior to classical linear methods for an easy and low-cost identification of MetS in patients treated with antipsychotics [28].

Following from this, the aim of this study was to assess whether an artificial neural network can be used to accurately predict MetS. The objective was to present an examination of the separate and simultaneous effects of a full range of predictive variables, including sleep and work-related stress variables, as well as clinical variables, to test ANN. In order to achieve an optimal ANN architecture, we considered ANN with different hidden layers and different numbers of neurons in each hidden layer.

## Methods

### Design and participants

Using a census design, 503 employees at an oil refinery in southern Iran in 2018 were invited to join the study. All had at least 1 year of work experience. Thirty-five people declined, yielding a final sample of 468 employees who gave informed consent. Data was collected in three stages: a survey instrument, anthropometric measurements, and biochemical measurements.

### Measures and procedure

Stage 1: A survey instrument was administered to collect demographic characteristics (age, sex, education level, and marital status), lifestyle habits (smoking, regular exercise, sleep duration), aspects of work time (job tenure, work hours, shift schedule) and measures of work-related stress and disturbed sleep.

- Smoking habit was defined as “current smoker” or “non-smoker” (never smoked / quit smoking > 1 year).
- Exercise habit was determined as participation in more than 30 min of moderate physical activity, twice a week, for over a year.
- National Sleep Foundation recommendations of 7–8 h [32] were used to categorise sleep duration as recommended or not.
- Work schedules outside of daytime hours were considered as shiftwork.
- Work-related stress was measured using the Management Standards Indicator Tool [11] which comprises 35-items across seven dimensions: Demands, Control, Management Support, Peer Support, Relationships, Role, and Change. All items were rated on a five-point Likert scale, and subscale scores calculated from averages. Low scores represented stressful working conditions, while high scores indicated a desirable situation. For each stressor participants were classified according to MSIT benchmarks (available at www.hse.gov.uk/stress): very desirable (≥80th percentile); desirable (≥50th percentile and < 80th percentile); undesirable (≥20th percentile and < 50th percentile); and very undesirable (<20th percentile). MSIT is an appropriate and valid measure for defining status in each category of working conditions [12]. The Persian language version used is valid and reliable [14]. In this study Cronbach alphas for the seven subscales were consistently good (range .77–.82).
- STOP-BANG [33] was used to screen for OSA. This questionnaire comprises eight dichotomous items associated with OSA: **S**noring, **T**iredness, **O**bserved apnoea during sleep, and high blood **P**ressure (STOP); **B**ody Mass Index> 35 kg/m^2^, **A**ge > 50 years, **N**eck circumference > 40 cm, and **G**ender (BANG). Three positive responses indicate a high risk of OSA. The Persian language version used is reliable and valid [34].

Stage 2: Anthropometric measurements.

Weight and height were measured using calibrated digital weighing scales and a rigid stadiometer; participants wore light clothing and no shoes. Body mass index (BMI) was calculated as weight (kg) divided by height (m^2^). Waist circumference was taken to the nearest 1 mm using a non-stretchable tape measure at the end of normal expiration, at the midpoint between the lower rib margin and the upper edge of the iliac crest, while participants stood in a relaxed position with arms at their sides. Blood pressure was measured using a calibrated standard mercury sphygmomanometer; each participant was measured twice using their right arm in a seated position after about 15 min of rest. An interval of at least one minute separated the two recordings; the average was used in analyses.

Stage 3: Biochemical measurements.

Baseline blood samples were collected from participants after 12-h of overnight fasting. Levels of serum glucose and lipid profile revolving around plasma triglyceride, total cholesterol, low-density lipoprotein cholesterol (LDL-C), and high-density lipoprotein cholesterol (HDL-C) were assayed using an enzymatic method kit.

### Diagnosis of metabolic syndrome

The diagnostic criterion for MetS followed the modified ATPІІІ definition [2]. Participants with at least three of the following five criteria were classified as having MetS:

- Obesity (waist circumference > 102 cm (Male), > 88 cm (Female))
- Hyperglycaemia (fasting plasma glucose≥100 mg/dl)
- Dyslipidaemia (triglyceride≥150 mg/dl)
- HDL-C < 40 mg/dl (Male), < 50 mg/dl (Female)
- Hypertension (blood pressure > 130 mmHg systolic, or > 85 mmHg diastolic).

### Statistical analyses

All analyses were conducted using SPSS software, version 22 (SPSS Inc., Chicago, IL, USA) and R-3.4 package. A *p*-value ≤ .05 represented statistical significance. Assumptions of normality were confirmed. To investigate the separate and simultaneous effects of predictive work-related risk factors and sleep variables on MetS as dependent variables alongside other predictors of MetS, a logistic regression with backward stepwise regression analysis was used and the variables that remained in the final model presented. Odds ratios (OR), with corresponding 95% confidence intervals, were used to show effect sizes in the model.

Not all methods of logistic regression can satisfactorily predict the result from non-linear relationships. Regression models can become increasingly complex as more and more variables are included in an analysis. Moreover, they can become excessively convoluted when details such as polynomials and interactions are explored. Hence, we chose to use a hierarchical logistic regression (HLR) methodology as a way to identify which predictors make a significant statistical contribution to MetS in our substantial model. Using a hierarchical regression allowed us to ascertain the variables that make a statistically significant contribution to explained variance in MetS after accounting for all other variables entered into the model.

### Artificial neural networks

Artificial neural networks (ANN) use computing systems to mimic the learning pattern of the highly interconnected neural networks in the human brain [35]. Whilst there are various types of ANN architecture [36], we used the multi-layer perceptron type because this methodology can be trained to approximate smooth measurable functions [37]. Multi-layer perceptron have been shown to be more effective that traditional statistical techniques [37]. They are set up to operate in a similar way to biological neural networks. That is, a natural neural wiring system has axons, dendrites and synapses, which enable communication via electric pulses. Depending on the strength of the pulse a neuron receives, it will produce an output signal and pass this through a synapse to the axon of a proximal neuron. The multi-layer perceptron is similarly a system of interconnected artificial neurons (perceptron) with nodes representing a non-linear mapping of an input layer and an output layer. The nodes are like switches that receive input from other nodes [35]. The weight of the switch corresponds to the multiplication of input by the node. Weights can be both excitatory and inhibitory. Output signals emerge as a function of the sum of inputs to a node and how they are modified by this non-linear activation function [37]. For us, the output is our estimate of the probability of the input as a predictor of MetS.

To prepare for implementing a regression artificial neural network (ANN), quantitative variables were scaled to fall on the closed [0,1] interval, and categorical variables were converted to dummy numeric variables. Age was coded 0 when ≤45 years and 1 for older; marital status was coded 0 for single and 1 for married; sex was coded 0 for female and 1 for male; job tenure was coded 0 for up to 15 years and 1 when longer; education was coded as 0 for High School level and 1 for higher levels. Shiftwork, exercise and smoking habits were coded 0 if “no” and 1 when “yes”.

As above, multilayer perceptron was used to develop ANN to model the presence of MetS in our sample of oil refinery workers. Multilayer perceptron architecture is variable, and generally it will include several layers. Thus, between the input and output layers there is one or more *hidden layers* which allow the ANN to transform the input space into *h* dimensions, where *h* is a number chosen by us. We then perform a logistic regression (sigmoid function) on this transformed space to estimate output.

We partitioned the data randomly into training and test subsets. Following convention and our experience, training data comprised 75% of the sample (*n* = 351) and the remaining 25% (*n* = 117) was used as test data. Artificial neural network training is about finding weights that allow accurate prediction (here of MetS). A challenge in training ANN is that too little training will underfit both the training and the test subsets, and too much training – indicated by a significant decrement in performance – means that the model is overfit, and this will negatively impact upon the test set. We used the Akaike Information Criterion, which penalises over fitting, to determine the best-fitting model. Unlike conventional statistical methods, several discretional elements are involved in building and training ANN. One of these is determining the basic network architecture, including the number of hidden layers and the number of neurons within each hidden layer. Although some texts propose that one hidden layer is usually sufficient to model complex nonlinear patterns, others argue for using ANN with more than one hidden layer. We believe this is an empirical question best addressed with experimentation so ANN with more than one hidden layer were evaluated in our study.

As is usual, ANN training started with a set of randomly generated weights, followed by backpropagation to update the weights towards accurately mapping all inputs to outputs. Backpropagation is simply an algorithm, commonly used for training ANN, to make an efficient search for the optimal weight values. We then formulated a complete backpropagation algorithm and tested that it worked in arbitrary feed-forward networks with differentiable activation functions at the nodes. To do this, we first computed a linear combination of the covariates (X), using some weight matrices *W* ∈ *R*^(*d* + 1) × *h*^_,_ where *d* denotes the dimension of the input variables and *h* is the number of neurons in the hidden layers. We set *z* = *XW*, then a logit function is applied to *z* (*σ*).

The hidden layer H can be considered a design matrix which contains the output of a logistic regression, and is able to classify each node according to whether it is activated or not: *h* = *σ*(*z*), and $$ H=\left[1\kern0.5em h\right]=\left[1\kern0.5em \sigma (z)\right]=\left[\begin{array}{cc}1& \sigma (XW)\end{array}\right] $$. For the output layer, we computed a linear combination of the hidden variables, this time using another weight matrix, *V* ∈ *R*^(*h* + 1) × (*k* − 1)^, where *k* is the number of possible classes (here *k* = 2). Then we applied one more function to get the output $\overset{\wedge }{MetS}=\sigma (u)$, where $u= HV=\left[1\kern0.5em h\right]V$. This is a probability vector, ${\overset{\wedge }{MetS}}_i=P\left({MetS}_i=1\right)$. When combined: ${\overset{\wedge }{MetS}}_i=\sigma (HV)=\sigma \left(\left[1\kern0.5em \sigma (XW)\right]V\right)$.

The main goal of our training was to reduce error in the network. In order to reduce error, we needed to change weights values. The log-likelihood for a binary classifier is: $l=\sum \limits_i\left({MetS}_i\log {\overset{\wedge }{MetS}}_i+\left(1-{MetS}_i\right)\log \left(1-{\overset{\wedge }{MetS}}_i\right)\right)$. We maximized this by using gradient descent, a general-purpose optimization algorithm. It calculates the gradient of the error function with respect to the weights within a specific neural network. The calculation proceeds backwards through the network: *W*_*t* + 1_ = *W*_*t*_ − *γ* ∇ *f*(*W*_*t*_), where *l* = *f*(*W*), *W*_*t*_ is the weight matrix at time *t*, ∇*f* is the gradient of *f* with respect to *W* and *γ* is the “learning” rate. Using the chain rule, the gradient of the log-likelihood with respect to the output weights is given by $\frac{\partial l}{\partial V}=\frac{\partial l}{\partial MetS}\frac{\partial MetS}{\partial V}$. The backpropagation algorithm calculated how much of the final output value is affected by each of the weights. To do this, calculations of partial derivatives were made, going back from the error function to the neuron that carried the specific weight.

We created ANN from 16 rounds of learning. Network architectures were varied by systematically reducing and increasing the number of neurons in the hidden layers. Each network used a backpropagation algorithm with sigmoid function as the nonlinear activation function in the hidden layers to predict the probability of the presence of MetS as an output. The training data supported learning by changing connection weights to subsequently generate predicted outcomes. In a process similar to cross-validation, the test data represented a holdout sample. Weights derived from training were applied to the test data and then predictions compared to what was known [19].

Results from ANN were compared to those from regression models using mean square error (MSE) acquired from predicted and observed values for test data. This approach provided assurance that the outcome was a valid representation [20].

## Results

Following ATPІІІ criteria [2] 37.6% participants were classified as having MetS. The association between the components of metabolic syndrome, and demographic and occupational variables, and MetS status via univariate analyses are shown in Table 1. Of the MetS components, BMI had the greatest impact on the prevalence of MetS as with an average increase of one unit of BMI, the risk of having MetS elevated 51%.

**Table 1:** Univariate comparisons of MetS components, demographic, occupational and lifestyle variables, according to MetS status (*n* = 468)

Variables		N (%)	Metabolic syndrome		*p* value	OR (95% CI)
			Absent (*n* = 292) Mean (±SD)	Present (*n* = 176) Mean (±SD)		
Waist circumference (cm)		468 (100)	92.90 (9.35)	105.90 (9.97)	<.001	1.15 (1.12–1.19)
Systolic blood pressure (mmHg)			119.12 (9.79)	127.47 (14.89)	<.001	1.06 (1.04–1.08)
Diastolic blood pressure (mmHg)			78.60 (5.90)	83.80 (12.82)	<.001	1.07 (1.04–1.10)
Fasting plasma glucose (mg/dl)			94.52 (17.83)	115.33 (26.28)	<.001	1.06 (1.04–1.07)
Plasma triglyceride (mg/dl)			134.07 (70.54)	214.87 (80.53)	<.001	1.01 (1.01–1.02)
HDL-C (mg/dl)			45.46 (11.37)	41.19 (8.14)	<.001	0.95 (0.93–0.97)
BMI (kg/m^2^)			26.02 (3.31)	30.44 (3.04)	<.001	1.51 (1.39–1.63)
Age^b^ (years)			40.31 (0.59)	46.03 (0.88)	<.001	1.09 (1.07–1.12)
Job tenure^b^ (years)			14.17 (0.63)	17.04 (0.30)	<.001	1.05 (1.02–1.08)
Working hours per Shift^b^			9.58 (0.76)	9.88 (0.17)	.77	1.02 (0.88–1.17)
Marital status^a^	Married	406 (86.8)	267 (65.8)	139 (34.2)	<.001	2.84 (1.64–4.91)
	Single	62 (13.2)	25 (40.3)	37 (59.7)		
Sex^a^	Male	398 (85)	267 (67.1)	131 (32.9)	<.001	3.66 (2.15–6.24)
	Female	70 (15)	25 (35.7)	45 (64.3)		
Education Level^a^	University degree	229 (48.9)	144 (62.9)	85 (37.1)	.83	1.04 (0.71–1.51)
	High school graduate	239 (51.1)	148 (61.9)	91 (38.1)		
Sleep time duration^a^	Recommended	320 (68.4)	209 (65.3)	111 (34.7)	.056	1.47 (0.99–2.80)
	Not as recommended	148 (31.6)	83 (56.1)	65 (43.9)		
Exercise habit^a^	Yes	300 (64.1)	218 (72.7)	82 (27.3)	<.001	3.37 (2.27–5.03)
	No	168 (35.9)	74 (44)	94 (56)		
Smoking habit^a^	Current smoker	98 (20.9)	31 (31.6)	67 (68.4)	<.001	0.19 (0.11–0.31)
	Non-smoker	370 (79.1)	261 (70.5)	109 (29.5)		
Shiftwork^a^	Yes	254 (54.3)	152 (59.8)	102 (40.2)	.215	0.78 (0.54–1.14)
	No	214 (45.7)	140 (65.4)	74 (34.6)		
STOP-BANG^a^	High risk	148 (31.6)	42 (28.4)	106 (71.6)	<.001	0.11 (0.07–0.17)
	Low risk	320 (68.4)	250 (78.1)	70 (21.9)		

^a^N (%)

^b^Mean (SD)

The mean age of participants was 42.46 ± 8.08 years (range: 28–65 years) and the ratio of female to male was 3:17. An increase of 1 year of age and 1 year of working experience, raised the risk of having MetS by 9 and 5%, respectively. Among the socio-demographic variables, marital status, sex, exercise and smoking habit were all significantly correlated with MetS status. STOP-BANG indicated that 31.6% of participants at high risk of OSA; the risk of developing MetS among those at low risk of OSA was 89% lower than those at high risk.

Regarding work-related stress, participants were categorized according to MSIT benchmarks and comparisons were made of those ≥80th percentile (very desirable) with those <20th percentile (very undesirable) on each of the seven dimensions of work-related stress. These two categories accounted for most participants’ self-reported stress levels, making a focus on these two levels appropriate. As reported in Table 2, the outcome of univariate analyses was a significant difference in the two levels for five of the seven WRS dimensions, strongly suggesting that WRS increases the risk of MetS.

**Table 2:** Frequency distribution and association with metabolic syndrome by MSIT stressor level (*N* = 468)

Stressor Level		N (%)	Metabolic syndrome		*p* value	OR (95% CI)
			Absent Mean(±SD)	Present Mean(±SD)		
Demand	very desirable	256 (54.7)	162 (63.3)	94 (36.7)	.18	1.32 (0.87–2.00)
	very undesirable	145 (31)	82 (56.6)	63 (43.4)		
Control	very desirable	70 (15)	58 (82.9)	12 (17.1)	<.001	4.73 (2.43–9.18)
	very undesirable	258 (60.9)	144 (50.5)	141 (49.5)		
Managerial support	very desirable	178 (38)	139 (78.1)	39 (21.9)	<.001	5.39 (3.40–8.55)
	very undesirable	186 (39.7)	74 (39.8)	112 (60.2)		
Peer support	very desirable	143 (30.6)	116 (81.1)	27 (18.9)	<.001	4.23 (2.62–6.84)
	very undesirable	280 (59.8)	141 (50.4)	139 (49.6)		
Relationships	very desirable	232 (49.6)	157 (67.7)	75 (32.2)	.20	1.36 (0.84–2.02)
	very undesirable	104 (22.2)	63 (60.6)	41 (39.4)		
Role	very desirable	160 (34.2)	134 (83.8)	26 (16.3)	<.001	5.67 (3.50–9.16)
	very undesirable	271 (57.9)	129 (47.6)	142 (52.4)		
Change	very desirable	311 (66.5)	240 (77.2)	71 (22.8)	<.001	7.32 (4.75–11.28)
	very undesirable	152 (32.5)	48 (31.6)	104 (68.4)		

A hierarchical logistic regression (HLR) model for multilevel analysis was used to determine the role of significant variables on MetS. The best result was obtained with the lowest mean squared error (MSE) value. Variables were entered into the HLR model in three stages: (1) demographic variables, (2) STOP-BANG and (3) MSIT, using backward stepwise binary logistic regression. As illustrated in Table 3, Step 3 is the model with the least AIC value, and the most adequate of our models. This included sex, one dimension of WRS – Role, and STOP-BANG (ie risk of OSA), as predictors of MetS.

**Table 3:** Factors associated with metabolic syndrome using hierarchical multivariate logistic regression (*n* = 351)

Characteristics	Step 1^a^	Step 2^b^	Step 3^c^
	OR (95% CI)	OR (95% CI)	OR (95% CI)
Sex	0.25 (0.13–0.49)**	0.09 (0.04–0.20)**	0.10 (0.04–0.23)**
Exercise habit	0.56 (−1.11 - -0.03)*	NS	NS
Smoking habit	3.01 (1.60–6.05)**	NS	NS
Age (years)	3.19 (1.88–5.47)**	NS	NS
STOP-BANG		3.74 (2.41–6.11)**	2.63 (1.57–4.54)**
Control			NS
Managerial support			NS
Peer support			NS
Role			0.55 (0.38–0.78)**
Change			NS
AIC	398.53	358.98	349.64

*NS* not significant

*p < .05, **p < .01

^a^corrected for age, job tenure, sleep time status, marital status, sex, exercise habit, smoker

^b^also corrected for STOP-Bang score

^c^and also corrected for dimensions of work-related stress

As described earlier, analysing data with ANN is an iterative process that involves experimentation with different network architectures and training parameters. To develop the ANN model, we performed 16 rounds of model learning using 17 input variables and one target variable. The best model, as indicated by lowest MSE, had 10 neurons in first hidden layer and 3 neurons in a second hidden layer (see Table 4).

**Table 4:** Comparison of predictive accuracy of artificial neural networks

Neural network	No. layers	Neurons in hidden layer(s)	MSE*1000
NN1	**2**	**(10,3)**	**105**
NN2	2	(10,2)	165
NN3	2	(9,3)	267
NN4	2	(9,2)	150
NN5	2	(8,3)	159
NN6	2	(8,2)	188
NN7	2	(7,3)	139
NN8	2	(7,2)	152
NN9	2	(6,3)	289
NN10	2	(6,2)	148
NN11	2	(5,3)	159
NN12	2	(5,2)	171
NN13	2	(4,3)	179
NN14	2	(4,2)	123
NN15	1	10	140
NN16	1	9	131

**Fig. 1:**
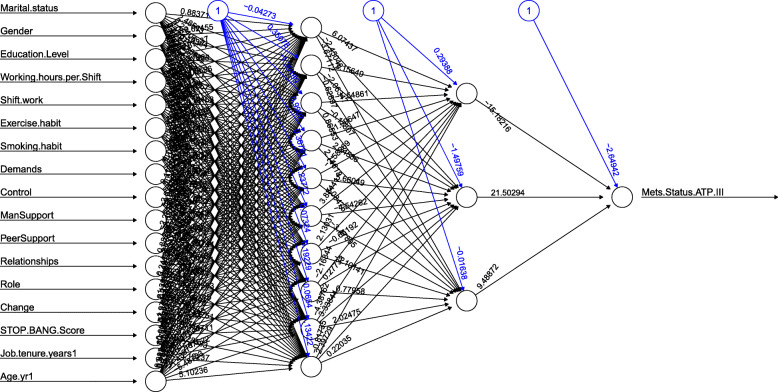
Plot of trained neural network including trained weights and basic information about the training process

We found networks with fewer neurons in the hidden layers hampered pattern recognition and reduced predictive accuracy, and networks with more neurons in the hidden layers captured patterns in training data that were not sustained on test data. After analysing the data, an appropriate neural network structure was achieved (see Fig. 1). The full set of weights of this neural network is presented in Additional file 1.

To compare results from HLR and ANN, we fitted HLR to the training data and computed MSE from the differentiation of the testing data and their predictive counterparts acquired from the fitted model. To compare the estimation errors of HLR and ANN, MSE, positive predictive values (PPV) and negative predictive values (NPV) were calculated. MSE for HLR was .28 whereas MSE for ANN was .105. To clarify these results, we also computed confusion matrices.

**Fig. 2:**
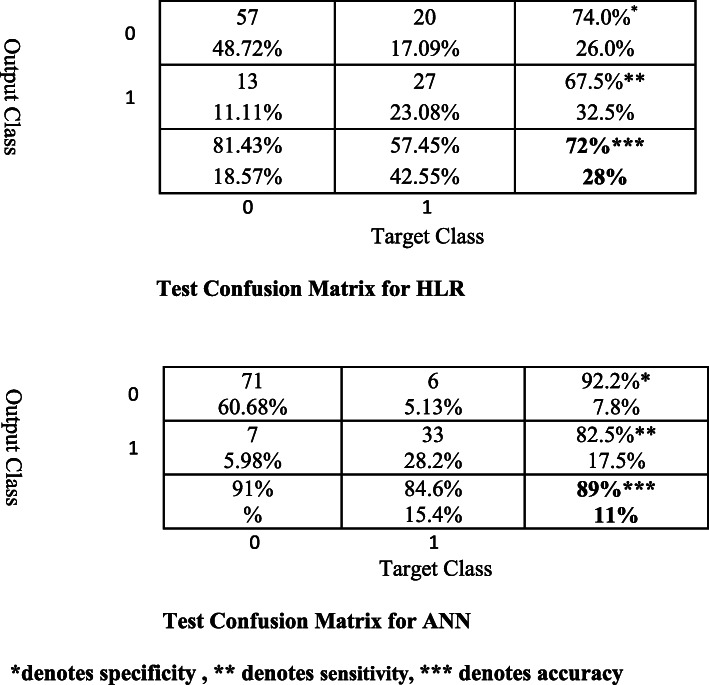
Test confusion matrices for HLR and ANN

The confusion matrices suggested 89% accuracy for ANN versus 72% accuracy for HLR in testing dataset (see Fig. 2). The ANN was able to detect 85% of participants who met the criteria for MetS whereas HLR detected only 57%. Regarding sensitivity, PPVs were 82.5% for the ANN, and 67.5% for HLR; regarding specificity, NPVs were 92.2% for the ANN system, and 74% for HLR.

## Discussion

Our findings show that ANN can be an effective tool for predicting MetS. We developed an ANN system that was able to capture functional relationships within data that included psychosocial workplace variables and disordered sleep variables, as well as anthropometric and biochemical clinical variables. Our results add to the literature in showing that work-related stress variables and OSA are important in the accurate identification of MetS status.

In line with previous literature [27, 29], we found ANN outperformed HLR analysis. In our study, HLR was powerful for *identifying* significant factors, but it did not perform well for *predicting* outcomes because its specificity was low. The ANN system had theoretical advantage over logistic regression and could effectively capturing non-linearity between the factors and the outcome. Interestingly, sensitivity and specificity of the ANN system were similar, suggesting that we had successfully generated appropriate predictions by applying ANN.

Over one third of our working-age participants had metabolic syndrome, according to ATPІІІ criteria [2]. This finding replicated the high prevalence of MetS found in previous studies in Iranian working populations [1, 3]. High rates of MetS are not confined to Iranian workers [1, 38–40]. In the United States [38], and in Korea [39], reports of higher prevalence rates in worker populations, implicate aspects of work as precipitators of MetS, even if different occupations present different levels of risk according to activity demands of the job [40].

Many studies have reported that abdominal obesity is a major risk factor for MetS and insulin resistance, as well as its association with dyslipidaemia, high blood pressure and hyperinsulinemia [1, 3, 5]. Our study similarly found higher BMI and greater waist circumference were associated with the probability of an increased incidence of MetS. There are mixed views on whether waist circumference or BMI is a better instrument for measuring obesity. In our results, the odds ratio for BMI was higher than waist circumference in determining the increased risk of MetS. Our results also replicate other studies which have found a high prevalence of other factors of MetS including blood pressure, fasting plasma glucose, plasma triglyceride and HDL-C [1, 3], and increased odds of MetS for females compared to males [1]. The sex difference can be partly attributed to the naturally higher prevalence of abdominal obesity and weight gain in females [41]. Similarly, our findings accord with other studies that have inferred an increasing trend towards developing of MetS with ageing [1, 2] which could be associated with the typical accumulation of fat in the abdominal area and increased insulin resistance as one ages [1, 5]. It has to be considered that age may also be an aspect of the greater risk of MetS we found in those with higher work experience.

Regarding lifestyle behaviour, MetS status was higher in those who smoked and did not exercise regularly. In this context, previous studies have identified that cigarette smoking increases triglycerides, lower HDL-C and hyperinsulinemia [30, 32]. Besides our study, there are other reports confirming that physical inactivity can strongly predict MetS [5, 40, 41] supporting assertions that the sedentary lifestyle of much of society is a causative factor in the high rates of MetS [4].

Work-related stress is a primary determinant of the health status of workers 10], and the relative risk of WRS positively predicts MetS [6]. Various measures of job stress have been used to verify this association, but to our knowledge this was the first study to refer to HSE’s Management Standards [9, 11] to clarify the impact of WRS on MetS. We found high levels of very undesirable working conditions, and that Control, Management Support, Peer Support, Role, and Change stressors independently increased the odds of having MetS. Dissatisfaction with working conditions is not unique to this workplace. Other population studies have found stressful aspects of work to play an important role in increasing the parameters of MetS [7, 40]. This can be explained through considering that the normally adaptive acute stress response is maladaptive in chronically stressful work situations through persistent triggering of the autonomic nervous system, and associated hormonal changes, glucose intolerance, and weight gain [41]. Role stress associated remained in the binary HLR model as independent predictors of MetS. Further exploring Role components in this workforce, we found that the information necessary for effective performance was ambiguous when presented to workers. In addition, the nature of their construction projects meant workers often faced changes to their work environment and ways of working. These observations add face validity to the findings and suggest focused strategies for stress management.

The impact of shiftwork, disturbed sleep, and OSA on MetS was another important focus of this study. Shiftwork is known to disrupt normal circadian rhythms and associated biological functions [15, 42–44]. Similarly, previous clinical and epidemiologic reports indicated that OSA presents a risk of developing MetS [16]. In agreement, we found a lower risk of OSA was associated with a lower probability of having MetS. However, we found no significant relevance of shiftwork to MetS, contrary to previous findings [42]. In this study, all participants worked the same number of hours regardless of whether on days or on shifts. Shiftwork comprised three rotating 8-h schedules. This meant no exposure to the longer hours of typical night shifts [43]. That said, in their systematic review, Canuto and colleagues, concluded that that evidence of an association of shiftwork and MetS was not robust [42]. They drew attention to variation in definitions of both shiftwork and MetS across studies. Critically, we cannot implicate 8-h shift schedules as a risk factor for MetS.

Two noteworthy strengths of this investigation were the recruitment of a community cohort, which permitted investigation of lifestyle and psychosocial workplace factors relatively cleanly, and the use of a robust measure of generic workplace stressors, which were found to contribute to MetS status, and offer a focus for intervention. The main limitation was its cross-sectional nature, and our inability to look at some potentially pertinent variables; these include the level of income and eating and drinking habits. As such, we recommend extension of the study to develop a deep understanding of causal relationships.

## Conclusions

Our performance evaluation of ANN indicated our model was highly efficient at predicting MetS. Following 16 rounds of model learning using 17 input variables and one target variable, we developed a model that had 10 neurons in first hidden layer and 3 neurons in a second hidden layer and was able to predict MetS with 89% accuracy. Based on our findings, preventive public health policies for reducing levels of MetS are necessary. These should focus on modifying lifestyle practices such as quitting smoking, having regular physical activity and an appropriate sleep schedule. There is also a strong case for effective risk assessment of WRS, and intervention where necessary. We also recommend screening opportunities in the workplace to detect early risk factors for MetS. There is a business case for this, as the high prevalence of MetS in working communities can be a major economic and operational burden. We assert that ANN are useful for identifying data patterns and those at risk of MetS, who can then be prioritised in terms of offering tests and interventions saving resources, including health.

## Supplementary Information

**Additional file 1.** Weights of ANN variables.

## Data Availability

The datasets used and analysed during the current study are available from either of the corresponding authors on reasonable request.

## Abbreviations

MetS: Metabolic syndrome
T2DM: Type 2 diabetes mellitus
ANN: Artificial neural network
OSA: Obstructive sleep apnea
WRS: Work-related stress
HSE: Health and Safety Executive
MSIT: Management Standards Indicator Tool
ATPIII: National Cholesterol Education Programme Adult Treatment Panel ІІI
BMI: Body mass index
LDL-C: low-density lipoprotein cholesterol
HDL-C: High-density lipoprotein cholesterol
HLR: Hierarchical logistic regression
MSE: Mean squared error
PPV: Positive predictive values
NPV: Negative predictive values
