# Supplementary material for: Prediction of metabolic syndrome based on sleep and work-related risk factors using an artificial neural network
Source: BMC Endocr Disord. 2020 Nov 12;20:169. doi: 10.1186/s12902-020-00645-x (PMC7659072; doi:10.1186/s12902-020-00645-x)
Supplement: Supplementary file 1 — Additional file 1. Weights of ANN variables. [file 12902_2020_645_MOESM1_ESM.docx]

| **Additional file 1**: Weights of ANN variables | |
| --- | --- |
| **Values** | **Parameters** |
| 4.003814468 | Error |
| 0.007452614 | reached.threshold |
| 364 | Steps |
| 442.0076289 | Aic |
| 1279.798239 | Bic |
| -0.042733241 | Intercept.to.1layhid1 |
| 0.883713668 | Marital.status.to.1layhid1 |
| -0.624554559 | Gender.to.1layhid1 |
| -0.872367816 | Education.Level.to.1layhid1 |
| 3.022173398 | Working.hours.per.Shift.to.1layhid1 |
| -0.035767952 | Shift.work.to.1layhid1 |
| -1.373679204 | Exercise.habit.to.1layhid1 |
| -3.788955435 | Smoking.habit.to.1layhid1 |
| 4.180870174 | Demands.to.1layhid1 |
| 0.117874035 | Control.to.1layhid1 |
| -2.031375089 | ManSupport.to.1layhid1 |
| -2.281584835 | PeerSupport.to.1layhid1 |
| 0.382941912 | Relationships.to.1layhid1 |
| 0.241350216 | Role.to.1layhid1 |
| -1.397091165 | Change.to.1layhid1 |
| -1.973130137 | STOP.BANG.Score.to.1layhid1 |
| -0.001025933 | Job.tenure.years1.to.1layhid1 |
| 0.98199612 | Age.yr1.to.1layhid1 |
| 0.350783513 | Intercept.to.1layhid2 |
| 2.485427928 | Marital.status.to.1layhid2 |
| 0.153635625 | Gender.to.1layhid2 |
| 0.719875732 | Education.Level.to.1layhid2 |
| -1.086963998 | Working.hours.per.Shift.to.1layhid2 |
| 0.669740237 | Shift.work.to.1layhid2 |
| 1.304411514 | Exercise.habit.to.1layhid2 |
| -6.703515756 | Smoking.habit.to.1layhid2 |
| -0.7958175 | Demands.to.1layhid2 |
| 1.14852808 | Control.to.1layhid2 |
| -3.867920385 | ManSupport.to.1layhid2 |
| -1.188739819 | PeerSupport.to.1layhid2 |
| -1.785528527 | Relationships.to.1layhid2 |
| 3.481277386 | Role.to.1layhid2 |
| 2.113095102 | Change.to.1layhid2 |
| -2.817249842 | STOP.BANG.Score.to.1layhid2 |
| -0.824246481 | Job.tenure.years1.to.1layhid2 |
| -3.85131895 | Age.yr1.to.1layhid2 |
| -1.30169203 | Intercept.to.1layhid3 |
| 0.224056847 | Marital.status.to.1layhid3 |
| -1.359850166 | Gender.to.1layhid3 |
| 0.58751083 | Education.Level.to.1layhid3 |
| 7.132673137 | Working.hours.per.Shift.to.1layhid3 |
| -1.191631564 | Shift.work.to.1layhid3 |
| -1.874276734 | Exercise.habit.to.1layhid3 |
| -0.477526457 | Smoking.habit.to.1layhid3 |
| 3.169600895 | Demands.to.1layhid3 |
| -1.032836908 | Control.to.1layhid3 |
| -5.205345556 | ManSupport.to.1layhid3 |
| -0.421902299 | PeerSupport.to.1layhid3 |
| 2.731758187 | Relationships.to.1layhid3 |
| -4.281475744 | Role.to.1layhid3 |
| 0.782394383 | Change.to.1layhid3 |
| -1.584943292 | STOP.BANG.Score.to.1layhid3 |
| 8.714807196 | Job.tenure.years1.to.1layhid3 |
| 0.153578822 | Age.yr1.to.1layhid3 |
| 0.990702834 | Intercept.to.1layhid4 |
| 0.066150634 | Marital.status.to.1layhid4 |
| -0.417550845 | Gender.to.1layhid4 |
| -3.685322025 | Education.Level.to.1layhid4 |
| 1.782106381 | Working.hours.per.Shift.to.1layhid4 |
| -2.598104732 | Shift.work.to.1layhid4 |
| 0.774433156 | Exercise.habit.to.1layhid4 |
| -5.947940786 | Smoking.habit.to.1layhid4 |
| -1.356227286 | Demands.to.1layhid4 |
| -2.748412918 | Control.to.1layhid4 |
| 1.67591143 | ManSupport.to.1layhid4 |
| 1.766439309 | PeerSupport.to.1layhid4 |
| 0.362043143 | Relationships.to.1layhid4 |
| -3.462313935 | Role.to.1layhid4 |
| -1.246099211 | Change.to.1layhid4 |
| 3.934086103 | STOP.BANG.Score.to.1layhid4 |
| -1.451813859 | Job.tenure.years1.to.1layhid4 |
| 3.242865376 | Age.yr1.to.1layhid4 |
| 0.367606079 | Intercept.to.1layhid5 |
| -3.013297589 | Marital.status.to.1layhid5 |
| -13.14473898 | Gender.to.1layhid5 |
| -0.064656678 | Education.Level.to.1layhid5 |
| 0.09769196 | Working.hours.per.Shift.to.1layhid5 |
| 0.666306933 | Shift.work.to.1layhid5 |
| 0.017874796 | Exercise.habit.to.1layhid5 |
| -4.324874736 | Smoking.habit.to.1layhid5 |
| 0.898560339 | Demands.to.1layhid5 |
| 0.452789095 | Control.to.1layhid5 |
| 1.701254112 | ManSupport.to.1layhid5 |
| -1.048699992 | PeerSupport.to.1layhid5 |
| -0.06803419 | Relationships.to.1layhid5 |
| -2.139630426 | Role.to.1layhid5 |
| -1.616926328 | Change.to.1layhid5 |
| 0.107877564 | STOP.BANG.Score.to.1layhid5 |
| 1.602292546 | Job.tenure.years1.to.1layhid5 |
| -0.180229609 | Age.yr1.to.1layhid5 |
| -1.237716432 | Intercept.to.1layhid6 |
| 0.353226637 | Marital.status.to.1layhid6 |
| -0.627471595 | Gender.to.1layhid6 |
| -2.05025483 | Education.Level.to.1layhid6 |
| 0.585293399 | Working.hours.per.Shift.to.1layhid6 |
| -0.17281835 | Shift.work.to.1layhid6 |
| 0.839587414 | Exercise.habit.to.1layhid6 |
| -0.955483391 | Smoking.habit.to.1layhid6 |
| -3.733987083 | Demands.to.1layhid6 |
| -3.122730373 | Control.to.1layhid6 |
| 1.207619065 | ManSupport.to.1layhid6 |
| 1.933681618 | PeerSupport.to.1layhid6 |
| 3.236213369 | Relationships.to.1layhid6 |
| 0.029307629 | Role.to.1layhid6 |
| 0.044135708 | Change.to.1layhid6 |
| 0.563120191 | STOP.BANG.Score.to.1layhid6 |
| 0.287929684 | Job.tenure.years1.to.1layhid6 |
| -0.627656663 | Age.yr1.to.1layhid6 |
| 0.073244799 | Intercept.to.1layhid7 |
| -1.082260963 | Marital.status.to.1layhid7 |
| -0.253494043 | Gender.to.1layhid7 |
| -0.945073995 | Education.Level.to.1layhid7 |
| -0.015294688 | Working.hours.per.Shift.to.1layhid7 |
| 0.721044286 | Shift.work.to.1layhid7 |
| 0.640218793 | Exercise.habit.to.1layhid7 |
| 10.57855315 | Smoking.habit.to.1layhid7 |
| 0.581127955 | Demands.to.1layhid7 |
| -2.134401124 | Control.to.1layhid7 |
| 2.849909623 | ManSupport.to.1layhid7 |
| -0.667436895 | PeerSupport.to.1layhid7 |
| -3.561821323 | Relationships.to.1layhid7 |
| 2.414731976 | Role.to.1layhid7 |
| 2.444627533 | Change.to.1layhid7 |
| -7.539732438 | STOP.BANG.Score.to.1layhid7 |
| 3.850151189 | Job.tenure.years1.to.1layhid7 |
| -2.15272864 | Age.yr1.to.1layhid7 |
| 0.192291291 | Intercept.to.1layhid8 |
| 0.565292703 | Marital.status.to.1layhid8 |
| -0.175679585 | Gender.to.1layhid8 |
| -0.915053442 | Education.Level.to.1layhid8 |
| 4.199461832 | Working.hours.per.Shift.to.1layhid8 |
| -1.643884673 | Shift.work.to.1layhid8 |
| -1.416803933 | Exercise.habit.to.1layhid8 |
| 1.649378882 | Smoking.habit.to.1layhid8 |
| 2.276727503 | Demands.to.1layhid8 |
| 4.944602698 | Control.to.1layhid8 |
| -7.607037807 | ManSupport.to.1layhid8 |
| -0.628303449 | PeerSupport.to.1layhid8 |
| 0.864531881 | Relationships.to.1layhid8 |
| -9.875828146 | Role.to.1layhid8 |
| -0.874749762 | Change.to.1layhid8 |
| -3.76233373 | STOP.BANG.Score.to.1layhid8 |
| 8.117827208 | Job.tenure.years1.to.1layhid8 |
| -1.43971207 | Age.yr1.to.1layhid8 |
| 0.065399199 | Intercept.to.1layhid9 |
| 1.057250802 | Marital.status.to.1layhid9 |
| -0.679769093 | Gender.to.1layhid9 |
| -2.595363347 | Education.Level.to.1layhid9 |
| 2.089650234 | Working.hours.per.Shift.to.1layhid9 |
| 0.824136361 | Shift.work.to.1layhid9 |
| 1.17775748 | Exercise.habit.to.1layhid9 |
| -1.230994593 | Smoking.habit.to.1layhid9 |
| 0.112813769 | Demands.to.1layhid9 |
| -2.152647504 | Control.to.1layhid9 |
| -0.524731265 | ManSupport.to.1layhid9 |
| -0.830285984 | PeerSupport.to.1layhid9 |
| 4.605002949 | Relationships.to.1layhid9 |
| 4.744980136 | Role.to.1layhid9 |
| -2.437506936 | Change.to.1layhid9 |
| -2.367112388 | STOP.BANG.Score.to.1layhid9 |
| -1.076223056 | Job.tenure.years1.to.1layhid9 |
| -5.487331437 | Age.yr1.to.1layhid9 |
| 1.134219903 | Intercept.to.1layhid10 |
| 3.083909092 | Marital.status.to.1layhid10 |
| 2.163951399 | Gender.to.1layhid10 |
| 6.104240693 | Education.Level.to.1layhid10 |
| 1.654340178 | Working.hours.per.Shift.to.1layhid10 |
| -4.155593498 | Shift.work.to.1layhid10 |
| 2.048221655 | Exercise.habit.to.1layhid10 |
| -1.138847621 | Smoking.habit.to.1layhid10 |
| -5.527327561 | Demands.to.1layhid10 |
| -8.388340132 | Control.to.1layhid10 |
| 1.703732955 | ManSupport.to.1layhid10 |
| -0.568639359 | PeerSupport.to.1layhid10 |
| -3.657085231 | Relationships.to.1layhid10 |
| -3.828728767 | Role.to.1layhid10 |
| 0.94631386 | Change.to.1layhid10 |
| 3.285328545 | STOP.BANG.Score.to.1layhid10 |
| 3.192365054 | Job.tenure.years1.to.1layhid10 |
| 5.102357547 | Age.yr1.to.1layhid10 |
| 0.29387611 | Intercept.to.2layhid1 |
| 6.074371947 | 1layhid.1.to.2layhid1 |
| 2.15648714 | 1layhid.2.to.2layhid1 |
| -1.548608622 | 1layhid.3.to.2layhid1 |
| -1.996468041 | 1layhid.4.to.2layhid1 |
| -2.582690403 | 1layhid.5.to.2layhid1 |
| 3.884408543 | 1layhid.6.to.2layhid1 |
| 2.130312463 | 1layhid.7.to.2layhid1 |
| -2.168435886 | 1layhid.8.to.2layhid1 |
| -4.387624217 | 1layhid.9.to.2layhid1 |
| -0.817350106 | 1layhid.10.to.2layhid1 |
| -1.497586718 | Intercept.to.2layhid2 |
| -2.450477061 | 1layhid.1.to.2layhid2 |
| -2.063471235 | 1layhid.2.to.2layhid2 |
| -0.178069522 | 1layhid.3.to.2layhid2 |
| 2.245363601 | 1layhid.4.to.2layhid2 |
| 5.660485357 | 1layhid.5.to.2layhid2 |
| -4.54262132 | 1layhid.6.to.2layhid2 |
| -0.661916859 | 1layhid.7.to.2layhid2 |
| 0.277334809 | 1layhid.8.to.2layhid2 |
| 3.338441264 | 1layhid.9.to.2layhid2 |
| 2.397289105 | 1layhid.10.to.2layhid2 |
| -0.016383287 | Intercept.to.2layhid3 |
| -3.671119073 | 1layhid.1.to.2layhid3 |
| -0.628973029 | 1layhid.2.to.2layhid3 |
| 0.865528207 | 1layhid.3.to.2layhid3 |
| 2.146179043 | 1layhid.4.to.2layhid3 |
| 3.084762984 | 1layhid.5.to.2layhid3 |
| -3.478945406 | 1layhid.6.to.2layhid3 |
| -1.101409826 | 1layhid.7.to.2layhid3 |
| 0.779578885 | 1layhid.8.to.2layhid3 |
| 2.024752562 | 1layhid.9.to.2layhid3 |
| 0.220349154 | 1layhid.10.to.2layhid3 |
| -2.649419542 | Intercept.to.Mets.Status.ATP.III |
| -15.18216006 | 2layhid.1.to.Mets.Status.ATP.III |
| 21.50293661 | 2layhid.2.to.Mets.Status.ATP.III |
| 9.488721267 | 2layhid.3.to.Mets.Status.ATP.III |
